# Outcomes of Rehabilitation Strategies for Pulmonary Atresia with Ventricular Septal Defect: A Single Center’s Experience

**DOI:** 10.31083/j.rcm2503084

**Published:** 2024-03-04

**Authors:** Shuai Zhang, Jianrui Ma, Xiang Liu, Tong Tan, Wen Xie, Haozhong Liu, Huimin Wang, Hailong Qiu, Shusheng Wen, Jimei Chen, Jian Zhuang, Haiyun Yuan, Jianzheng Cen

**Affiliations:** ^1^Guangdong Provincial Key Laboratory of South China Structural Heart Disease, 510080 Guangzhou, Guangdong, China; ^2^Department of Cardiovascular Surgery, Guangdong Cardiovascular Institute, Guangdong Provincial People’s Hospital, Guangdong Academy of Medical Sciences, 510080 Guangzhou, Guangdong, China; ^3^Shantou University Medical College, 515041 Shantou, Guangdong, China; ^4^Department of Radiology, Guangdong Cardiovascular Institute, Guangdong Provincial People’s Hospital, Guangdong Academy of Medical Sciences, Southern Medical University, 510080 Guangzhou, Guangdong, China

**Keywords:** pulmonary atresia, rehabilitation, shunt, right ventricle to pulmonary artery connection, outcome, complete repair

## Abstract

**Background:**

Both systemic-to-pulmonary shunt and right ventricle-pulmonary artery (RV-PA) connection are extensively applied to initially rehabilitate the pulmonary artery in pulmonary atresia with the ventricle septal defect (PA/VSD). However, which of these options is the most ideal for promoting pulmonary artery development and improving outcomes remains controversial.

**Methods:**

A total of 109 PA/VSD patients undergoing initial rehabilitative surgery at Guangdong Provincial People’s Hospital from 2010 to 2020 were enrolled in this study. A series of clinical data were collected to compare the perioperative and postoperative outcomes between systemic-to-pulmonary and RV-PA connection.

**Results:**

The mean duration of follow-up was 61.1 months in the systemic-to-pulmonary shunt group and 70.3 months in the RV-PA connection group (*p* > 0.05). The RV-PA connection technique resulted in a significantly higher ${\mathrm{PaO}}_{2}$, lower red blood cells (RBC), lower hemoglobin, and lower hematocrit (Hct) (*p* < 0.05). The cumulative incidence curve estimated a cumulative complete repair rate of 56 ± 7% after 5 years in the RV-PA connection group, significantly higher than 36 ± 7% after 5 years in the systemic-to-pulmonary shunt group (*p* < 0.05). The Kaplan-Meier curve revealed a similar estimated survival rate between the two groups (*p* = 0.73). The RV-PA connection was identified as an independent predictor for complete repair in the multivariable analysis (HR = 2.348, 95% CI = 1.131–4.873).

**Conclusions:**

The RV-PA connection is a more ideal initial rehabilitative technique than systemic-to-pulmonary shunt in treating PA/VSD as a consequence of comparable probability of survival but improved definitive complete repair rate.

## 1. Introduction

Pulmonary atresia with ventricular septal defect (PA/VSD) is a complex and unusual form of congenital heart lesion that is estimated to occur in 7 of 100,000 live births [1]. It is characterized by a pulmonary artery size ranging from absent/diminutive to reasonable, a tetralogy-type ventricular septal defect (VSD), and heterogeneous pulmonary blood supply generated from isolation or combination of major aortopulmonary collateral arteries (MAPCAs) and native pulmonary arteries. Prognosis of this condition is poor, with approximately one-fifth of patients only surviving to the age of 30 years [2]. The surgical management of this lesion has evolved for decades but remains challenging including two opposed strategies: MAPCAs unifocalization and pulmonary vessel rehabilitation.

The rehabilitation strategy aims to promote the development of native pulmonary arteries for definitive completer repair. It can be categorized into several subgroups according to the initial operative technique, such as the establishment of systemic-to-pulmonary shunt and right ventricle-pulmonary artery (RV-PA) connection. Both are beneficial for native pulmonary growth and eventual complete repair and are therefore extensively employed in many institutions [3, 4, 5, 6]. However, the ideal rehabilitative surgical option for treating patients with PA/VSD is highly debated.

In this paper, we sought to summarize our 11-year experience and describe our single-center outcomes of the RV-PA connection and systemic-to-pulmonary shunt in PA/VSD patients [7, 8].

## 2. Materials and Methods

### 2.1 Study Population and Data Collection

The present study was approved by the Guangdong Provincial People’s Hospital ethics committee following the ethical guidelines of the Declaration of Helsinki (1975). From January 2010 to December 2020, a total of 109 PA/VSD patients who underwent the initial rehabilitative surgery at Guangdong Provincial People’s Hospital were enrolled in this study. Those PA/VSD patients who received a unifocalization strategy rather than a rehabilitative strategy were excluded. Those combined with other complex congenital lesions such as transposition of the great arteries and dextrocardia were also excluded. Fifty-three patients underwent RV-PA connection, while fifty-six underwent systemic-to-pulmonary shunt. The choice of rehabilitative strategies was mainly determined by experienced surgeons in our center. Nonetheless, the following preferences were taken into consideration. Systemic-to-pulmonary shunting was performed in patients with poor native pulmonary artery growth within the pericardium ≤2.5 mm or in neonates and infants with age <3 months to avoid cardiopulmonary bypass. RV-PA connection were utilized in those with native pulmonary arteries >2.5 mm or in older infants. All patients’ medical history, preoperative testing, computed tomography (CT), operation records, and follow-up data were reviewed and collected. The McGoon ratio and Nakata index were calculated on CT imaging, as previously described [9].

### 2.2 Rehabilitation Technique

The included patients adopted rehabilitative strategies in terms of a right ventricle-to-pulmonary artery connection or a systemic-to-pulmonary artery shunt, which have been explicitly described before [10]. Briefly, in the context of the right ventricle to pulmonary artery connection, a median sternotomy was performed, followed by the establishment of cardiopulmonary bypass. The autologous pericardial graft, bovine pericardial grafts, bovine jugular vein, or Gore-Tex conduit would be used to widen or reconstruct the right ventricle to pulmonary artery connection. In patients adopting a systemic-to-pulmonary artery shunt, a central shunt by anastomosis of the aorta and the main pulmonary artery or a modified Blalock-Taussing shunt by anastomosis of the innominate artery and the ipsilateral branch pulmonary artery would be performed in the presence or absence of the cardiopulmonary bypass.

### 2.3 MAPCAs Management 

Angiography and more often CT would be performed preoperatively to determine the MAPCAs’ origin, size, supply, and distribution. The MAPCAs that were accompanied by serious stenosis or were the only source of pulmonary blood would be untreated. In contrast, those that were the sole pulmonary blood flow would be ligated during the rehabilitative surgery simultaneously or percutaneous occluded preoperatively. If heart failure or severe pulmonary hypertension occurs during the postoperative follow-up, percutaneous occlusion might also be considered.

### 2.4 Complete Repair

If there is a satisfactory growth of the pulmonary artery with the McGoon ratio >1.2~1.5 and the Nakata index >150 mm/$m^{2}$, a complete repair would be performed. The right ventricle outflow would be reconstructed with a valved conduit or an autologous patch, followed by a VSD closure. Flow monitoring would also be performed during the surgery. If pulmonary perfusion flow didn’t reach 3 L/(min⋅$m^{2}$) or pulmonary artery pressure more than 25 mmHg, a VSD patch fenestration would be created to maintain the hemodynamic stability. Those MAPCAs not providing the sole pulmonary blood supply and leading to significant stenosis would be also ligated as much as possible during the biventricular complete repair.

### 2.5 Definition and Follow-Up 

In-hospital morbidity was defined as the isolation or combination of the following postoperative complications at the hospital: extracorporeal membrane oxygenation support, delayed sternal closure, diaphragm plication, pneumonia, or cerebrovascular issues. In-hospital mortality referred to postoperative death before discharge. Late mortality was defined as post-discharge death either before or after the next stage of surgery. ΔMcGoon ratio/ΔNakata index was defined as the difference between the McGoon ratio/Nakata index before the complete repair and before the initial rehabilitative surgery.

All patients were requested for outpatient visits at 3, 6, and 12 months after the initial rehabilitative surgery and annually thereafter. The endpoints were follow-up death or complete repair. Therefore, we primarily compared the long-term outcomes in terms of survival and complete repair between two rehabilitative strategies in the overall PA/VSD cohort. Given more heterogenicity in PA/VSD with MAPCAs (PA/VSD/MAPCAs) patients, we also compared the outcomes targeting this sub-cohort.

### 2.6 Statistics

Continuous variables with normal distribution and abnormal distribution were shown as means with standard deviations and medians with ranges, respectively. Categorical variables were shown as frequencies with percentages. Student’s test or Mann-Whitney test was used to compare the continuous variables between two groups as appropriate. Chi-squared or Fisher’s exact test was adopted in categorical variables. The probability of survival by time was estimated by the Kaplan-Meier curve and compared between the two groups using the Log-rank test. The cumulative complete repair by time was estimated by the cumulative incidence curve and compared between the two groups using Gray’s test. The subdistribution hazards model was used to identify factors associated with complete repair (death as a competing event). Those factors with a *p* value less than 0.1 in the univariable competing risk regression analysis and those clinically significant factors were included in the multivariable analysis. Given few positive events, cox regression multivariable analysis for mortality was not performed. A two-sided *p* value less than 0.05 was considered significant. All statistical analysis were performed using IBM SPSS Statistics for Windows, version 26.0 (IBMCorp., Armonk, Westchester County, NY, USA) and R (R Core Team, 2022).

## 3. Results

### 3.1 Baseline Characteristics of PA/VSD Patients

**Fig. 1.**
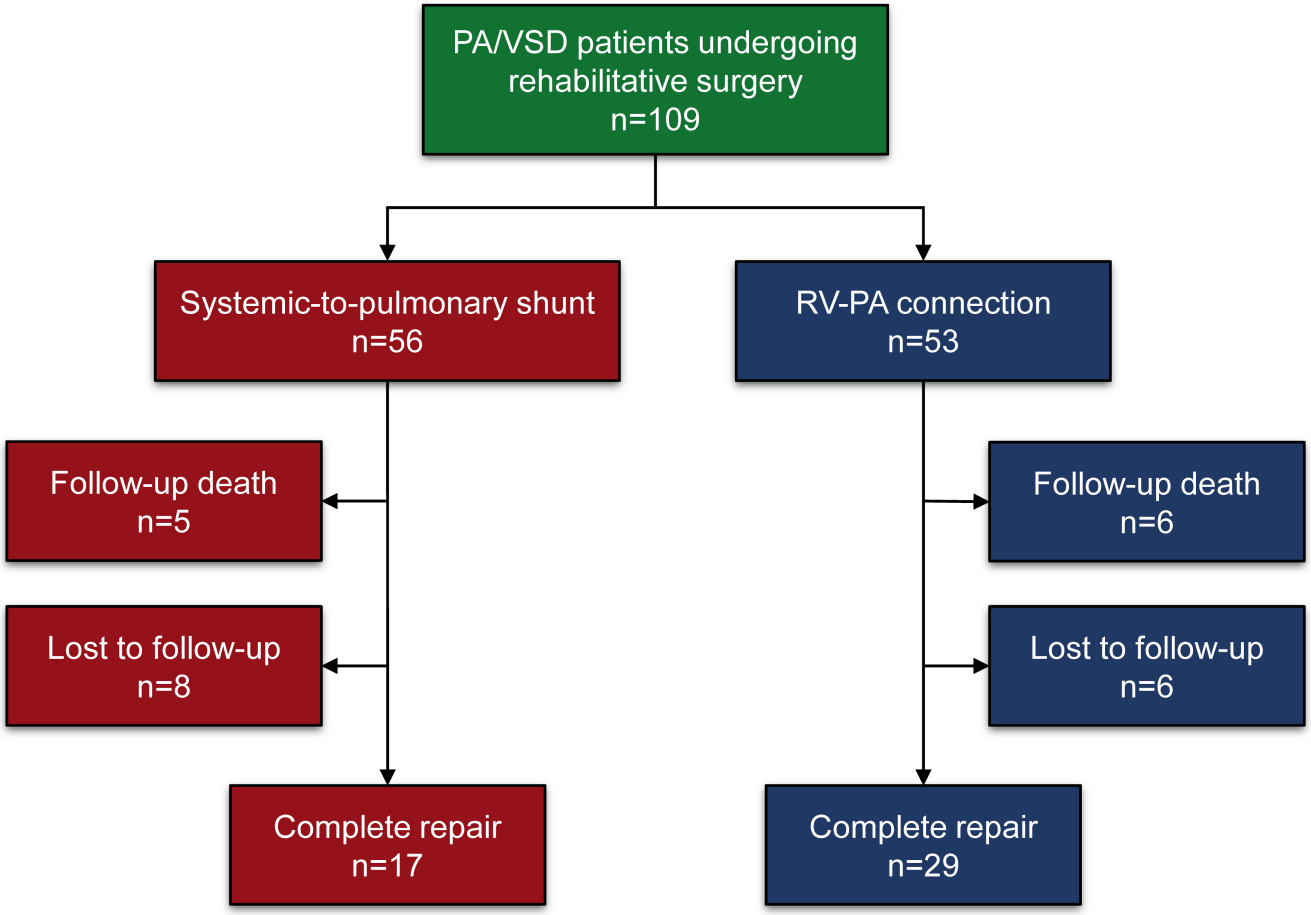
**Flowchart of rehabilitative technique options and outcomes in the overall PA/VSD patients cohort in our center**. PA/VSD, pulmonary atresia with the ventricular septal defect; RV-PA connection, right ventricle-pulmonary artery connection.

Overall, there were approximately 10–15 rehabilitative surgeries every year but less than 5 in 2019 and 2020. In this study, there were 17 patients who underwent complete repair surgery, with 5 deaths and 8 lost to follow-up among the systemic-to-pulmonary shunt group. In contrast, 29 patients were repaired completely with 6 deaths and 6 lost to follow-up among 53 patients receiving the initial RV-PA connection (Fig. 1). The baseline characteristics of the overall PA/VSD cohort and PA/VSD/MAPCAs sub-cohort are summarized in Table 1. In the overall PA/VSD cohort, the McGoon ratio in the RV-PA connection was significantly larger than that in the systemic-to-pulmonary shunt (*p* = 0.003). The other preoperative data were distributed evenly between the two groups. In the PA/VSD/MAPCAs sub-cohort, there was also a significantly larger McGoon ratio and lower levels of hemoglobin and hematocrit (Hct) in the RV-PA connection group (*p* < 0.05). No significant differences regarding other preoperative data were observed between the two groups.

**Table 1.** **Baseline characteristics of overall PA/VSD cohort and PA/VSD/MAPCAs sub-cohort**.

	Overall PA/VSD cohort			PA/VSD/MAPCAs sub-cohort		
	Systemic-to-pulmonary shunt	RV-PA connection	*p*-value	Systemic-to-pulmonary shunt	RV-PA connection	*p*-value
	n = 56	n = 53		n = 25	n = 22	
Gender, male	30 (53.6%)	32 (60.4%)	0.473	10 (40.0%)	8 (36.4%)	0.798
Age (month)	12.0 (1.6–85.7)	12.8 (4.5–48.2)	0.739	66.0 (10.8–222.9)	32.4 (16.1–68.1)	0.316
Height (cm)	71.0 (51.0–112.0)	72.0 (59.5–95.0)	0.783	115.0 (70.5–151.0)	86.0 (73.5–101.0)	0.166
Weight (kg)	8.0 (3.6–16.0)	8.0 (5.3–12.4)	0.966	18.0 (8.0–44.0)	11.0 (8.0–13.8)	0.086
BSA ($m^{2}$)	0.39 (0.20–0.74)	0.40 (0.27–0.59)	0.804	0.78 (0.39–1.35)	0.49 (0.41–0.65)	0.138
${\mathrm{PaO}}_{2}$ (%)	73 (66–80)	73 (66–80)	0.930	73 (71–81)	77 (72–84)	0.375
RBC	6.1 ± 1.5	6.2 ± 1.5	0.800	6.4 ± 1.2	6.0 ± 1.1	0.209
Hemoglobin (g/L)	168.0 ± 35.2	159.4 ± 32.2	0.130	177.6 ± 37.4	157.2 ± 21.7	0.025
Hct (%)	0.52 ± 0.11	0.49 ± 0.10	0.122	0.54 ± 0.12	0.47 ± 0.07	0.022
ALT (μ/L)	18.0 (13.0–27.6)	17.5 (14.0–22.8)	0.572	18.0 (12.5–27.0)	15.0 (13.8–21.0)	0.387
TBil (μmol/L)	14.9 (10.4–23.1)	14.6 (9.0–21.0)	0.238	16.3 (10.1–23.4)	12.2 (9.1–20.6)	0.301
DBil (μmol/L)	4.5 (3.0–6.3)	4.6 (3.2–6.4)	0.734	3.9 (2.8–5.4)	4.1 (3.2–5.9)	0.670
Scr (μmol/L)	32.0 (23.0–43.0)	30.9 (22.0–36.5)	0.337	37.0 (23.9–55.0)	28.2 (22.7–34.3)	0.070
PT (sec)	14.1 ± 1.5	13.8 ± 1.0	0.119	14.3 ± 1.6	13.7 ± 0.8	0.143
Presence of MAPCAs	25 (44.6%)	22 (41.5%)	0.741	25 (100%)	22 (100%)	/
Patent ductus arterious	21 (38.5%)	22 (41.5%)	0.669	6 (24.0%)	7 (31.8%)	0.550
McGoon ratio	0.79 ± 0.31	0.99 ± 0.37	0.003	0.66 ± 0.33	0.91 ± 0.38	0.020
Nakata index (${\mathrm{mm}}^{2}$/$m^{2}$)	74.1 ± 45.2	61.3 ± 38.0	0.114	57.7 ± 42.8	54.5 ± 36.4	0.789

PA/VSD, pulmonary atresia with ventricular septal defect; PA/VSD/MAPCAs, pulmonary atresia with ventricular septal defect and major aortopulmonary collateral arteries; RV-PA connection, right ventricle-pulmonary connection; BSA, body surface area; MAPCAs, major aortopulmonary collateral arteries; RBC, red blood cells; Hct, hematocrit; ALT, alanine aminotransferase; TBil, total bilirubin; DBil, direct bilirubin; Scr, serum creatine; PT, prothrombin time.

### 3.2 Comparison of Perioperative Outcome

As shown in Table 2, the cardiopulmonary bypass (CPB) time and aortic cross-clamp time when adopting systemic-to-pulmonary shunt were all 0 min, which was significantly shorter than when adopting the RV-PA connection (*p* < 0.001). The median size of the shunt/conduit was 10.0 mm in the RV-PA connection group, significantly bigger than 5.0 mm in the systemic-to-pulmonary shunt group (*p* < 0.001). The RV-PA connection resulted in a significantly higher ${\mathrm{PaO}}_{2}$. No significant difference was observed between the two groups regarding the other perioperative variables. There were 0 deaths during hospitalization in both groups. In the PA/VSD/MAPCAs sub-cohort, there was also a significantly bigger shunt/conduit and a higher postoperative ${\mathrm{PaO}}_{2}$ in the RV-PA connection group (*p* < 0.05).

**Table 2.** **Perioperative outcome between two rehabilitative techniques in the overall PA/VSD cohort and PA/VSD/MAPCAs sub-cohort**.

	Overall PA/VSD cohort			PA/VSD/MAPCAs sub-cohort		
	Systemic-to-pulmonary shunt	RV-PA connection	*p*-value	Systemic-to-pulmonary shunt	RV-PA connection	*p*-value
	n = 56	n = 53		n = 25	n = 22	
CPB time (min)	0 (0–0)	107 (86.0–138.5)	<0.001	0.00 (0.00–0.00)	103.5 (86.5–145.8)	<0.001
Aortic cross-clamp time (min)	0 (0–0)	62 (44.0–84.0)	<0.001	0 (0–0)	61.0 (44.5–76.3)	<0.001
Shunt/conduit size (mm)	5.0 (4.0–6.0)	10.0 (10.0–12.0)	<0.001	6.0 (5.0–7.5)	10.0 (9.5–10.0)	<0.001
Postoperative ${\mathrm{PaO}}_{2}$ (%)	85 (80–90)	90 (85–95)	<0.001	85 (82–89)	90 (85–95)	0.005
Mechanical ventilation time (h)	57.7 (26.1–118.1)	55.8 (27.8–140.2)	0.132	53.9 (23.8–92.1)	51.2 (25.9–108.6)	0.848
In-hospital morbidity	10 (17.9%)	7 (13.2%)	0.504	5 (20.0%)	2 (9.1%)	0.524
In-hospital mortality	0	0	/	0	0	/
Hospital stay (d)	28.0 (21.3–40.5)	33.0 (24.0–47.5)	0.482	28.0 (21.0–39.5)	33.5 (23.8–41.8)	0.153

PA/VSD, pulmonary atresia with ventricular septal defect; PA/VSD/MAPCAs, pulmonary atresia with ventricular septal defect and major aortopulmonary collateral arteries; RV-PA connection, right ventricle-pulmonary connection; CPB, cardiopulmonary bypass.

### 3.3 Long-Term Outcome after the Initial Rehabilitative Surgery

In the overall cohort, the mean duration of follow-up in the systemic-to-pulmonary shunt and RV-PA connection group was 61.1 ± 31.4 months and 70.3 ± 26.8 months, respectively, as shown in Table 3 (*p* > 0.05). The levels of red blood cells (RBC), hemoglobin, and Hct were all significantly higher in those with initial systemic-to-pulmonary shunts compared to those with an initial RV-PA connection (*p* < 0.001). The RV-PA connection would contribute to a significantly larger ΔMcGoon ratio (*p* < 0.001) and ΔNakata index (*p* < 0.001). There was no significant difference regarding inter-stage intervention before complete repair including pulmonary angioplasty, pulmonary stenting, and MAPCAs coil occlusion between the two groups. However, shunt/conduit replacement was more frequent in the systemic-to-pulmonary shunt group than in the RV-PA connection (*p* < 0.001). Similar follow-up results to the overall cohort were also shown in the PA/VSD/MAPCAs sub-cohort.

**Table 3.** **Follow-up results after the initial rehabilitative surgery in the PA/VSD cohort and PA/VSD/MAPCAs sub-cohort**.

		Overall PA/VSD cohort			PA/VSD/MAPCAs sub-cohort		
		Systemic-to-pulmonary shunt	RV-PA connection	*p*-value	Systemic-to-pulmonary shunt	RV-PA connection	*p*-value
		n = 56	n = 53		n = 25	n = 22	
Duration of follow-up (month)		61.1 ± 31.4	70.3 ± 26.8	0.103	62.7 ± 28.8	70.6 ± 28.1	0.349
RBC		5.9 (5.3–6.8)	5.3 (4.8–5.9)	<0.001	5.7 (5.3–6.6)	5.3 (4.8–6.1)	0.021
Hemoglobin (g/L)		156.0 (145.0–179.5)	140.0 (130.0–154.6)	<0.001	161.0 (150.5–177.5)	139.0 (128.2–157.0)	<0.001
Hct (%)		0.48 (0.44–0.55)	0.42 (0.39–0.47)	<0.001	0.52 (0.46–0.56)	0.43 (0.37–0.47)	<0.001
ΔMcGoon ratio		0.41 (0.24–0.64)	0.69 (0.43–1.04)	<0.001	0.33 (0.24–0.56)	0.57 (0.35–0.94)	0.017
ΔNakata index (${\mathrm{mm}}^{2}$/$m^{2}$)		57.8 (26.8–92.7)	131.3 (94.5–232.4)	<0.001	49.7 (27.9–85.0)	111.4 (56.1–144.8)	0.006
Inter-stage intervention							
	Pulmonary angioplasty	2 (3.6%)	2 (3.8%)	>0.999	0	0	/
	Pulmonary stenting	1 (1.8%)	1 (1.9%)	>0.999	0	0	/
	MAPCAs coil occlusion	0	1 (1.9%)	0.978	0	1 (4.5%)	0.948
	Shunt/conduit replacement	20 (35.7%)	2 (3.8%)	<0.001	9 (36.0%)	1 (4.5%)	0.023
Late mortality		5 (8.9%)	6 (11.3%)	0.679	1 (4.0%)	3 (13.6%)	0.511
Complete repair		17 (30.4%)	29 (54.7%)	0.010	2 (8.0%)	11 (50.0%)	0.001

PA/VSD, pulmonary atresia with ventricular septal defect; PA/VSD/MAPCAs, pulmonary atresia with ventricular septal defect and major aortopulmonary collateral arteries; RV-PA connection, right ventricle-pulmonary connection; MAPCAs, major aortopulmonary collateral arteries; RBC, red blood cells; Hct, hematocrit.

**Fig. 2.**
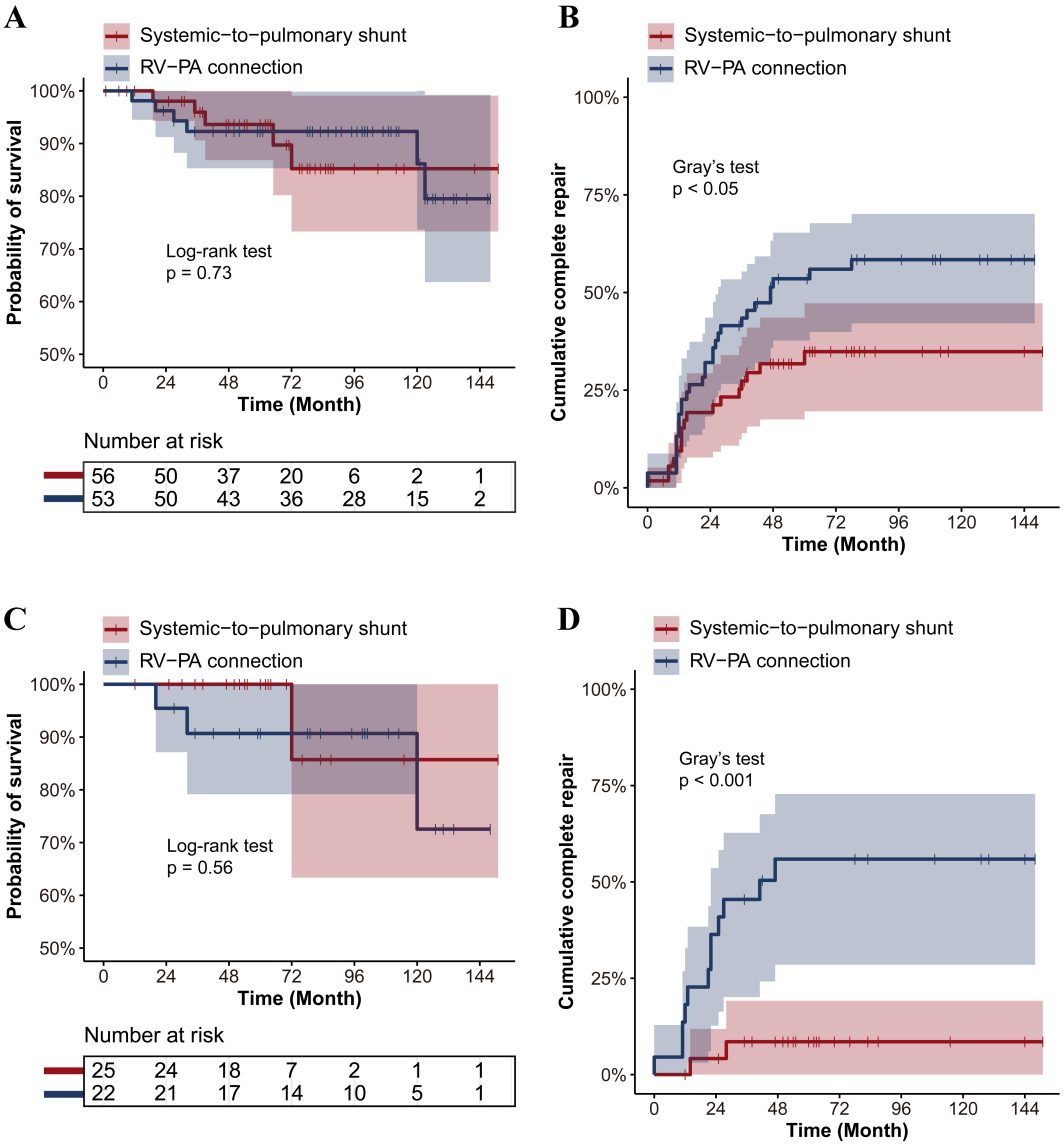
**Long-term outcomes between the two rehabilitative surgeries in the overall PA/VSD cohort (A,B) and the PA/VSD/MAPCAs sub-cohort (C,D)**. (A) No significant difference regarding the probability of survival by the time between the two rehabilitative surgeries in the overall PA/VSD cohort (*p* > 0.05). (B) RV-PA connection showed a significantly higher cumulative complete repair rate by time than the systemic-to-pulmonary shunt in the overall PA/VSD cohort (*p* < 0.05). (C) No significant difference regarding the probability of survival by the time between the two rehabilitative surgeries in the PA/VSD/MAPCAs sub-cohort (*p* > 0.05). (D) RV-PA connection showed a significantly higher cumulative complete repair rate by time than the systemic-to-pulmonary shunt in the PA/VSD/MAPCAs sub-cohort (*p* < 0.001). PA/VSD, pulmonary atresia with ventricular septal defect; PA/VSD/MAPCAs, pulmonary atresia with ventricular septal defect and major aortopulmonary collateral arteries; RV-PA connection, right ventricle-pulmonary artery connection.

**Fig. 3.**
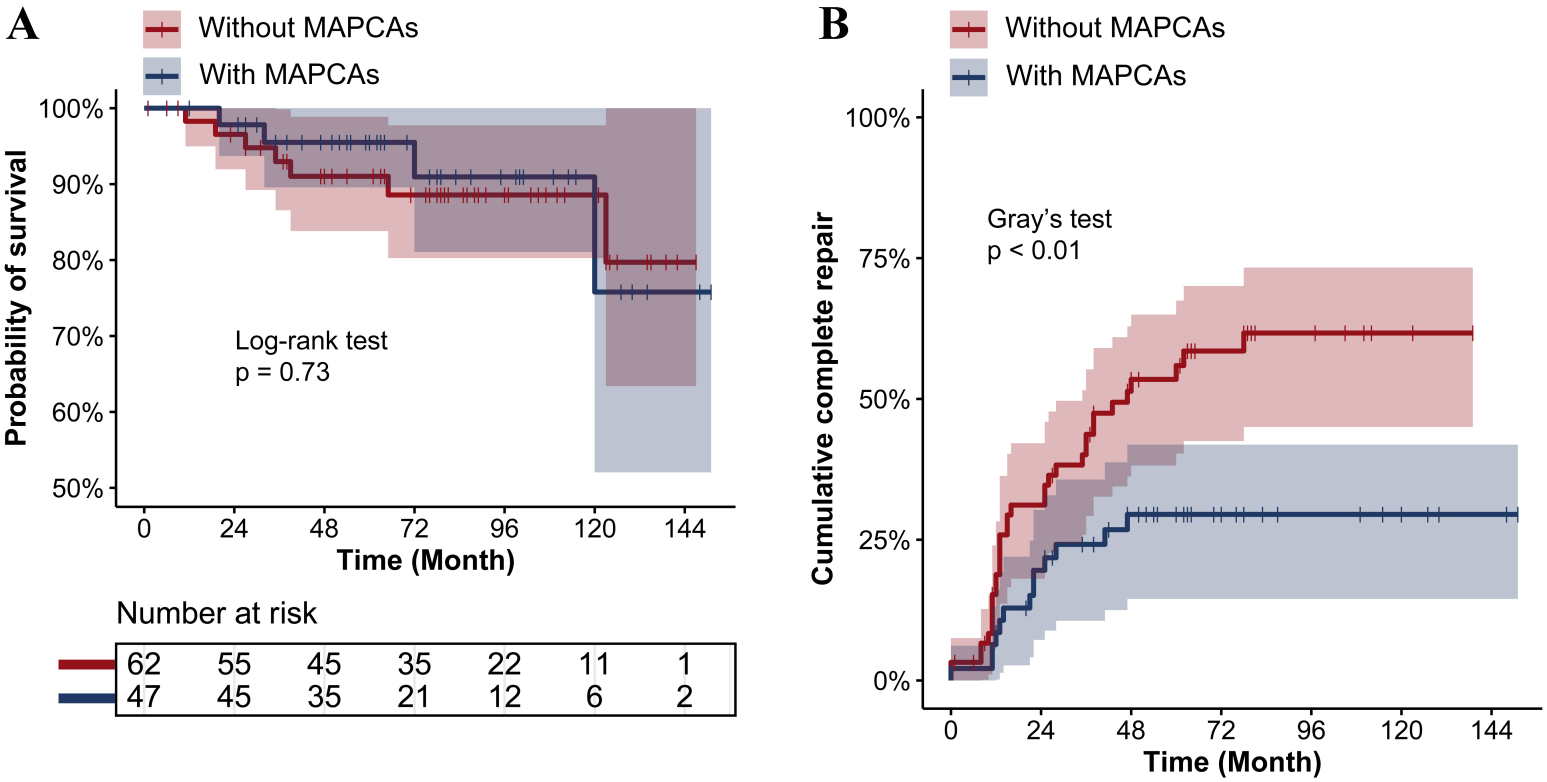
**Long-term outcomes between PA/VSD patients without MAPCAs and PA/VSD/MAPCAs patients**. (A) No significant difference regarding the probability of survival by the time between PA/VSD patients without MAPCAs and PA/VSD/MAPCAs patients (*p* > 0.05). (B) PA/VSD patients without MAPCAs showed a significantly higher cumulative complete repair rate by time than PA/VSD/MAPCAs patients (*p* < 0.01). PA/VSD, pulmonary atresia with ventricular septal defect; PA/VSD/MAPCAs, pulmonary atresia with ventricular septal defect and major aortopulmonary collateral arteries; MAPCAs, major aortopulmonary collateral arteries.

The late mortality in the two groups was similar with no significant difference in either the overall PA/VSD and PA/VSD/MAPCAs sub-cohort. In the overall cohort, there were 5 deaths in the systemic-to-pulmonary shunt group and 6 deaths in the RV-PA connection group. In the systemic-to-pulmonary shunt group, one patient died of multiple organ dysfunction syndrome after 35 months and one patient died of cardiogenic shock after 19 months. Two patients died of pneumonia after 65 and 72 months. In the RV-PA connection group, one death occurred due to myocarditis after 32 months. The cause of other sudden deaths in the two groups was unknown. Compared with those undergoing systemic-to-pulmonary shunt, patients with the RV-PA connection showed a significantly higher incidence of complete repair in either the overall PA/VSD and PA/VSD/MAPCAs sub-cohort (*p* < 0.05). Specifically, 17 patients (30.4%) in the systemic-to-pulmonary shunt group and 29 patients (54.7%) in the RV-PA connection group achieved complete repair during the follow-up in the overall PA/VSD cohort. In the PA/VSD/MAPCAs sub-cohort, there were 11 patients (50.0%) with RV-PA connection and 2 patients (8.0%) with systemic-to-pulmonary shunts eventually undergoing complete repair. Fig. 2 showed the probability of survival and cumulative complete repair by time between the two rehabilitative strategies in the overall PA/VSD cohort and the PA/VSD/MAPCAs sub-cohort. The probability of survival rate was similar between the two groups in the overall PA/VSD cohort (*p* = 0.73). A significantly higher cumulative complete repair rate was observed in the RV-PA connection group than in the systemic-to-pulmonary shunt group (*p* < 0.05). The cumulative complete repair rate in the RV-PA connection group was estimated to be 56 ± 7% after 5 years, in contrast to 36 ± 7% in the systemic-to-pulmonary shunt group. In the PA/VSD/MAPCAs sub-cohort, no significant difference regarding the probability of survival between the two groups was observed (*p* = 0.73). The RV-PA connection resulted in a significantly higher cumulative complete repair rate than the systemic-to-pulmonary shunt (*p* < 0.001). The 5-year complete repair rates were 54 ± 11% in the RV-PA connection group, in contrast to 8 ± 6% in the systemic-to-pulmonary shunt. In Fig. 3, similarly, no significant difference regarding the survival probability between PA/VSD patients without MAPCAs and PA/VSD/MAPCAs patients was observed (*p* < 0.05). However, the cumulative complete repair rate in the PA/VSD patients without MAPCAs was estimated to be 59 ± 7% after 5 years, significantly higher than 30 ± 7% in the PA/VSD/MAPCAs patients.

### 3.4 Multivariable Analysis for Complete Repair

In a univariable competing risk regression analysis, age, presence of MAPCAs, Patent ductus arteriouos, and RV-PA connection were identified as predictors for complete repair. These factors combined with clinically significant factors (${\mathrm{PaO}}_{2}$, McGoon ratio, and Nakata index) were entered into the multivariable analysis. Eventually, age (hazard ratio (HR) = 0.990, 95% confidence interval (CI) = 0.980–0.999) and RV-PA connection (HR = 2.348, 95% CI = 1.131–4.873) were identified as independent predictors for completer repair (*p* < 0.05) (Table 4). Given the limited number of patients as well as few positive events, cox regression multivariable analysis for complete repair was not performed on the PA/VSD/MAPCAs sub-cohort.

**Table 4.** **Univariable and multivariable analysis for complete repair in the overall PA/VSD cohort**.

	Univariable analysis		Multivariable analysis	
	HR (95% CI)	*p*-value	HR (95% CI)	*p*-value
Age	0.989 (0.981–0.998)	0.012	0.990 (0.980–0.999)	0.039
${\mathrm{PaO}}_{2}$	0.981 (0.959–1.004)	0.108	0.994 (0.970–1.018)	0.627
Presence of MAPCAs	0.425 (0.224–0.808)	0.009	1.269 (0.611–2.638)	0.523
Patent ductus arterious	2.035 (1.139–3.637)	0.016	0.599 (0.327–1.096)	0.096
McGoon ratio	1.569 (0.721–3.414)	0.257	0.331 (0.084–1.295)	0.112
Nakata index	1.003 (0.997–1.010)	0.309	1.009 (0.997–1.019)	0.154
RV-PA connection	1.914 (1.050–3.487)	0.034	2.348 (1.131–4.873)	0.022

PA/VSD, pulmonary atresia with ventricular septal defect; RV-PA connection, right ventricle-pulmonary connection; MAPCAs, major aortopulmonary collateral arteries; HR, hazard ratio; 95% CI, 95% confidence interval.

## 4. Discussion

This study summarized the 11-year experience of PA/VSD patients’ outcomes between two different initial rehabilitative strategies at our center over the past decade. The main findings are shown as follows: (1) Compared with the RV-PA connection, the systemic-to-pulmonary shunt would result in a lower ${\mathrm{PaO}}_{2}$ and higher level of RBC, hemoglobin, and Hct. (2) The RV-PA connection was associated with a higher cumulative complete repair rate and comparable survival rate.

The ${\mathrm{PaO}}_{2}$ immediately after the initial RV-PA connection was significantly higher than that after the systemic-to-pulmonary shunt. The comparison of oxygenation saturation immediately after the two rehabilitative strategies is rarely reported. The difference might be predominantly attributed to the more sufficient antegrade pulmonary blood flow provided by the RV-PA connection. In contrast to the systemic-to-pulmonary shunt, this strategy allows maintaining diastolic pressure as well as directing adequate desaturated blood into the lungs in an antegrade manner. Regardless of the VSD left open, the streaming effect will also direct a large proportion of systemic venous blood flowing via the conduit, which results in a more satisfactory oxygenation saturation [11]. Therefore, the elevated level of RBC, hemoglobin, and Hct are assumed as a compensated reaction in the context of a long-term relatively desaturated systemic-to-pulmonary shunt.

RV-PA connection resulted in a significantly higher cumulative completer repair rate than the systemic-to-pulmonary shunt despite the similar impact on promoting pulmonary vasculature development. The multivariable analysis in this study showed that RV-PA connection was an independent predictor for complete repair (HR = 2.348, 95% CI = 1.131–4.873). The previous investigation on the postoperative outcome of PA/VSD patients undergoing two different rehabilitative surgeries remains controversial. Fan *et al*. [7] reviewed and compared 44 patients undergoing systemic-to-shunt and 54 patients undergoing RV-PA connection from 2011 to 2016 at their center, showing that the pulmonary vessel growth, as well as complete repair rate, were similar between these two groups. In their study, the mean age of systemic-to-pulmonary shunt and RV-PA connection was 25.0 months and 27.6 months, respectively, much older than our patients. They underwent the initial surgery late, in part probably due to their relatively stable condition. In this context, the impact of both these two rehabilitative strategies on pulmonary artery growth and complete repair might not be distinct. In contrast, Zhao also compared the outcome of these two rehabilitative strategies, enrolling a total of 157 PA/VSD/MAPCAs patients from 2009 to 2017. They found that the RV-PA connection was more beneficial for improving the cumulative complete repair rate, which is consistent with our findings [8]. Multiple reasons could explain the better potential of the RV-PA connection in promoting pulmonary vasculature development and final complete repair in our study. First of all, the RV-PA connection directs the aforementioned sufficient blood flow into the lungs in an antegrade and pulsatile manner, beneficial for pulmonary vasculature development. Indeed, more frequent replacement of shunt/conduit that usually aimed to provide more adequate blood flow occurred in the systemic-to-pulmonary shunt group than in the RV-PA connection group. It also allows pulmonary artery intervention like balloon angioplasty via antegrade access. Secondly, the systemic-to-pulmonary shunt was associated with pulmonary artery distortion, overcirculation, and thrombosis [12]. Shunt thrombosis and obstruction occurred in several systemic-to-pulmonary shunt patients in our study, which might aggregate the oxygenation desaturation and hinder pulmonary artery growth. Thirdly, the mean McGoon ratio in the RV-PA connection group was higher, indicating an initially better pulmonary vessel size, which probably in part contributed to our findings. The higher McGoon index and pulmonary artery confluence have been demonstrated to be capable of improving the probability of complete repair [13].

The previous major studies on PA/VSD patients who initially underwent rehabilitative surgery were summarized in Table 5 (Ref. [3, 6, 7, 8, 13, 14, 15, 16, 17, 18, 19, 20, 21]). The complete repair rate in either the overall PA/VSD cohort or PA/VSD sub-cohort was slightly lower than the previous investigation [6, 14, 22], which is somewhat attributed to the following reasons. First, patients (median age: 12.8 months) in this study were older than those in most of the previous studies, as evidenced by Table 5. Some patients were referred to hospitals late in our country mainly owing to their family’s poor financial situation, limited pediatric specialists, insufficient bed availability, and mild cyanosis. It has been suggested that the initial rehabilitative surgery performed in PA/VSD patients with age more than approximately half a year old appeared to reduce the probability of complete repair [23]. Secondly, regular outpatient clinic visits after the initial rehabilitative surgery are imperative for the assessment of pulmonary vasculature growth, timely inter-stage intervention, and eventual complete repair. However, proportional patients did not strictly follow the schedule because of misconceptions about PA/VSD disease, the long distance from home to the hospital, and the family’s poor financial situation, which may have compromised the likelihood of complete repair. Thirdly, the mean period of follow-up was 70.3 months in the RV-PA connection group and 61.1 months in the systemic-to-pulmonary shunt group, which is relatively short compared to some previous studies [15, 16]. Of note, the complete repair rate in the PA/VSD/MAPCAs patients was particularly lower than that in the previous studies, compared with PA/VSD patients without MAPCAs. We assumed that PA/VSD/MAPCAs had a worse dysplasia of pulmonary artery than PA/VSD without MAPCAs so that proportional PA/VSD/MAPCAs were unable to reach satisfactory pulmonary vasculature and achieve complete repair in the context of mere rehabilitative strategy. Hence, the adoption of a unifocalization strategy or combined strategy for these patients is also practical and important [11]. The management of PA/VSD/MAPCAs remains highly challenging because of great heterogeneity. More future studies focusing on individual choice of treatment strategies (rehabilitation, unifocalization, or combination) for PA/VSD/MAPCAs to improve long-term outcomes are warranted.

**Table 5.** **Summary of main studies on PA/VSD patients initially undergoing rehabilitative surgery**.

Author	Years of study	Patient number	Age on surgery	Rehabilitation technique	Follow-up period	Survival	Complete repair
1. Macalister *et al*. [15]	1989–2019	107	7 d (4–26)	SPS	10.5 y (3.6–18.8)	Estimated 10-year survival rate: 81%	85%
2. Zhao *et al*. [8]	2009–2017	56	13.9 m (1.8–211.5)	Central shunt	18 m (0–66)	92.9%	Estimated 5-year complete repair rate: 56.0 ± 11.6%
		56	10.4 m (2.6–216.9)	RV-PA connection	22 m (0–62)	91.1%	Estimated 5-year complete repair rate: 74.5 ± 7.2%
3. Fan *et al*. [7]	2011–2016	44	25.0 ± 31.5 m	SPS	11.4 ± 10 m	97.7%	20.5%
		54	27.6 ± 49.3 m	RV-PA connection	15.5 ± 11.8 m	88.9%	37%
4. Zou *et al*. [17]	2010–2019	29	8 m (0.5–144)	SPS	24 m (6–116)	Estimated 10-year survival rate: 76.1%	54.2%
		68	14 m (2.2–209.6)	RV-PA connection	47 m (22–222)		50%
5. Chen *et al*. [6]	2009–2014	69	1.8 ± 1.8 y	RV-PA connection	2.8 ± 1.3 y	5-year survival rate: 93.8 ± 3.0%	Estimated 3-year complete repair rate: 60.1 ± 7.1%
6. Alsoufi *et al*. [18]	2002–2012	58	6 d (3–11)	B-T shunt	5.1 ± 3.9 y	Estimated 8-year survival rate: 72.2%	79.3%
7. Choi *et al*. [19]	2011–2015	13	1.8 ± 1.8 d	RV-PA connection	26.0 ± 16.8 m	84.6%	76.9%
8. Bradley *et al*. [14]	2004–2007	10	9 d (4–86)	RV-PA connection	1.9 ± 0.9 y	90%	80%
9. Hofbeck *et al*. [13]	1976–1988	104	218.3 d	Rehabilitation (not detailed)	4.95 y (2 d–13.75 y)	Estimated 10-year survival rate: 69%	36.5%
10. Soquet *et al*. [20]	2003–2014	33	3.3 w (0.4–31.9)	Central shunt (most frequent)	4.5 y	90.9%	73%
11. Lee *et al*. [21]	2004–2017	50	22 d (16.0–36)	Modified B-T shunt (most frequent)	59.3 m (22–115)	Estimated 5-year survival rate: 83.6%)	86%
12. Kim *et al*. [3]	1993–2013	15	1.91 m (0.2–26.36)	Central shunt	70.7 ± 67.1 m	Estimated 5-year survival rate: 82.5%	92.9%
13. Kaskinen *et al*. [16]	1970–2007	109	0.96 m	SPS (most frequent)	11.4 y (0.01–41.77)	Estimated 5-year survival rate: 66%	50.0%

PA/VSD, pulmonary atresia with ventricular septal defect; SPS, systemic-to-pulmonary shunt; RV-PA connection, right ventricle-pulmonary artery connection; B-T shunt, Blalock-Taussig shunt; d, day; w, week; m, month; y, year.

There are several limitations in this study. First, the retrospective nature and single-center design may limit its generalization to other centers. The initial pulmonary vasculature between groups was different, which at least, in part, influenced the final comparison of pulmonary growth and complete repair rate. Therefore, these findings should be assessed with caution.

## 5. Conclusions

The rehabilitative strategies in terms of RV-PA connection and systemic-to-pulmonary shunt resulted in a similar survival rate in PA/VSD patients. The RV-PA connection is more advantageous as an initial rehabilitative technique than the systemic-to-pulmonary shunt to improve the eventual complete repair rate.

## Data Availability

Data are available from the corresponding author upon reasonable request.

## Abbreviations

PA/VSD, pulmonary atresia with ventricular septal defect; PA/VSD/MAPCAs, pulmonary atresia with ventricular septal defect and major aortopulmonary collateral arteries; RV-PA connection, right ventricle-pulmonary artery connection; VSD, ventricular septal defect; MAPCAs, major aortopulmonary collateral arteries; CT, computed tomography; B-T shunt, Blalock-Taussig shunt.
